# Megasphaera *elsdenii* and *Saccharomyces cerevisiae* as direct fed microbials and their impact on ruminal microbiome during an acute acidosis challenge in continuous culture

**DOI:** 10.1093/tas/txad123

**Published:** 2023-10-21

**Authors:** Jose A Arce-Cordero, Ting Liu, Hugo F Monteiro, Kwang C Jeong, Antonio P Faciola

**Affiliations:** Escuela de Zootecnia, Universidad de Costa Rica, San Jose, 11501-2060, Costa Rica; Emerging Pathogens Institute, University of Florida, Gainesville, FL 32611, USA; Department of Population Health and Reproduction, School of Veterinary Medicine, University of California, Davis, CA 95616, USA; Emerging Pathogens Institute, University of Florida, Gainesville, FL 32611, USA; 3 Department of Animal Sciences, University of Florida, Gainesville, FL 32611, USA

**Keywords:** in vitro, lactate, propionate, succinate, yeast

## Abstract

Our objective was to evaluate the effects of combinations of *Saccharomyces cerevisiae* and *Megasphaera elsdenii* as direct-fed microbials (DFM) on ruminal microbiome during an acute acidosis challenge in a continuous culture system. Treatments provided a DFM dose of 1 × 10^8^ colony-forming unit (CFU)/mL, as follows: control (no DFM), YM1 (*S. cerevisiae* and *M. elsdenii* strain 1), YM2 (*S. cerevisiae* and *M. elsdenii* strain 2), and YMM (*S. cerevisiae* and half of the doses of *M. elsdenii* strains 1 and 2). We conducted four experimental periods of 11 d, which consisted of non-acidotic days (1 to 8) and acidotic challenge days (9 to 11) to establish acute ruminal acidosis conditions with a common basal diet containing 12% neutral detergent fiber and 58% starch. Treatments were applied from days 8 to 11, and samples of liquid and solid-associated bacteria were collected on days 9 to 11. Overall, 128 samples were analyzed by amplification of the V4 region of bacterial 16S rRNA, and data were analyzed with R and SAS for alpha and beta diversity, taxa relative abundance, and correlation of taxa abundance with propionate molar proportion. We observed a lower bacterial diversity (Shannon index, *P* = 0.02) when YM1 was added to the diet in comparison to the three other treatments. Moreover, compared to control, addition of YM1 to the diet increased relative abundance of phylum *Proteobacteria* (*P* = 0.05) and family *Succinivibrioceae* (*P* = 0.05) in the solid fraction and tended to increase abundance of family *Succinivibrioceae* (*P* = 0.10) and genus *Succinivibrio* (*P* = 0.09) in the liquid fraction. Correlation analysis indicated a positive association between propionate molar proportion and relative abundance of *Proteobacteria* (*r* = 0.36, *P* = 0.04) and *Succinivibrioceae* (*r* = 0.36, *P* = 0.05) in the solid fraction. The inclusion of YM1 in high-grain diets with a high starch content resulted in greater abundance of bacteria involved in succinate synthesis which may have provided the substrate for the greater propionate synthesis observed.

## Teaser

Combination of live yeasts *Saccharomyces cerevisiae* with lactate-utilizing bacteria *Megasphaera elsdenii* may increase propionate synthesis through a greater abundance of succinate synthesizing bacteria.

## Introduction

High rate of inclusion of grain in beef cattle diets is a well-known factor that favors acute ruminal acidosis (Owens et al., 1998). As detailed below, previous studies have demonstrated that supplementing direct-fed microbials (DFM) can aid with amelioration of ruminal conditions during acidosis. Specifically, the role of lactate-consuming bacteria such as *Megasphaera elsdenii (M. elsdenii*), which is a common inhabitant of the rumen, consists of utilizing the excess of lactate resulting from starch fermentation to synthesize propionate (Nocek et al., 2002). Such a process is known as the acrylate pathway (Ungerfeld, 2020) and some estimates indicate that up to 80% of lactate utilization for propionate synthesis is carried out by *M. elsdenii* (Counotte et al., 1981).

Supplementation with yeast, specifically *Saccharomyces cerevisiae* (*S. cerevisiae*) has been found to increase ruminal pH and decrease lactate concentration in ruminal fluid (Desnoyers et al., 2009), which has been attributed to the oxygen-scavenging role of yeasts allowing a more reduced ruminal environment (McAllister et al., 2011) which may favor lactate-utilizing bacteria (Yoon and Stern, 1995). Previous work suggests possible benefits of DFM combinations on animal response. Combinations of *S. cerevisiae* and lactate-producing bacteria, have been shown to increase digestion of corn silage and ruminal pH in cows consuming diets containing 70% grain (Nocek et al., 2002), and greater ruminal digestibility of corn silage and milk production in cows consuming typical dairy rations (Nocek and Kautz, 2006).

In our companion study (Monteiro et al., 2022a) we simulated acute ruminal acidosis in vitro using a dual-flow continuous culture system and evaluated the impact of including combinations of *S. cerevisiae* and two strains of *M. elsdenii* as DFM with high-grain diets on ruminal fermentation. We observed that inclusion of DFM resulted in a greater molar proportion of propionate in the ruminal fluid when compared to the control, suggesting a stimulation of propionate-synthesizing bacteria as a result of DFM supplementation.

Our present study aims to evaluate the effects of supplementing combinations of *S. cerevisiae* and two strains of *M. elsdenii* as DFM with a high-grain diet on ruminal microbiome. Based on the results of our companion study (Monteiro et al., 2022a) we hypothesized that inclusion of DFM would have an impact on bacterial populations associated with propionate synthesis.

## Materials and Methods

Animal care and handling procedures required for this study were conducted under protocols that were approved by Institutional Animal Care and Use Committee at the University of Florida, in accordance with the Guide for the Care and Use of Agricultural Animals in Research and Teaching (FASS, 2010).

### Experimental Design and Treatments

A detailed description of the design and treatments can also be found in our companion study (Monteiro et al. 2022a). Briefly, eight fermenters of a dual-flow continuous culture system were arranged in a duplicated 4 × 4 Latin square design with treatments defined by combinations of additives added to a common basal diet as shown in Table 1. Each period consisted of 11 d of fermentation, which included a first phase with a non-acidotic diet (days 1 to 8) followed by a high-grain acidotic challenge diet (days 9 to 11) to establish acute ruminal acidosis conditions. Diets for the experiment were formulated according to NASEM (2016) recommendations. Ingredients for experimental diets were ground to 2 mm particle size in a Wiley mill (model N°2; Arthur H. Thomas Co., Philadelphia, PA) and a subsample of 500 g from each ingredient was ground to 1 mm for chemical analyses.

**Table 1.** Ingredient and chemical composition of experimental diets

Item	Treatment^1^			
	Control	YM1	YM2	YMM
Item, %DM				
Non-acidotic diet (days 1 to 8)				
Alfalfa hay	50.0	50.0	50.0	50.0
Ground corn	45.1	45.1	45.1	45.1
Soybean meal	1.9	1.9	1.9	1.9
Mineral and vitamin premix	3.0	3.0	3.0	3.0
Challenge diet (days 9 to 11)				
Alfalfa hay	10.0	10.0	10.0	10.0
Ground corn	78.4	78.4	78.4	78.4
Soybean meal	8.60	8.60	8.60	8.60
Mineral and vitamin premix	3.00	3.00	3.00	3.00
Additive, mL/d (days 8 to 11)				
Carrier^2^	2.00	0.00	0.00	0.00
* Saccharomyces cerevisiae* ^3^	0.00	1.00	1.00	1.00
* Megasphaera Elsdenii* strain 1^4^	0.00	1.00	0.00	0.50
* Megasphaera Elsdenii* strain 2^5^	0.00	0.00	1.00	0.50
Chemical composition, %DM^6^				
CP	13.5	13.5	13.5	13.5
NDF	12.0	12.0	12.0	12.0
ADF	5.67	5.67	5.67	5.67
Starch	57.8	57.8	57.8	57.8
TDN	80.6	80.6	80.6	80.6

^1^Treatments based on direct-fed microbials (DFM) supplementation: CTRL, carrier of additives without DFM; YM1, *Saccharomyces cerevisiae* and *Megasphaera elsdenii* strain 1; YM2, *Saccharomyces cerevisiae* and *Megasphaera elsdenii* strain 2; YMM, *Saccharomyces cerevisiae* and *Megasphaera elsdenii* strain 1 (1/2 dose) and strain 2 (1/2 dose).

^2^Aqueous solution of 15% glycerol.

^3^Solution of *Saccharomyces cerevisiae* (1 × 10^8^ colony-forming unit (CFU)/mL) in 15% glycerol carrier.

^4^Solution of *Megasphaera elsdenii* strain 1 (1 × 10^8^ CFU/mL) in 15% glycerol carrier.

^5^Solution of *Megasphaera elsdenii* strain 2 (1 × 10^8^ CFU/mL) in 15% glycerol carrier.

^6^Composition of challenge diet (days 9 to 11): DM (dry matter), CP (crude protein), NDF (neutral detergent fiber), ADF (acid detergent fiber); TDN (total digestible nutrients).

Treatments were applied at a rate of 2 mL per day, divided into two equal 1 mL doses that were infused into each fermenter from days 8 to 11 at the moment of morning and evening feeding. A detailed description of treatments is provided in Table 1, which shows a constant concentration of 1 × 10^8^ colony-forming unit (CFU)/mL for all DFM treatments. The following DFM combinations were evaluated: CTRL (no DFM, containing only the carrier: aqueous solution of 15% glycerol), YM1 (the carrier plus *S. cerevisiae* and *M. elsdenii* strain 1), YM2 (the carrier plus *S. cerevisiae* and *M. elsdenii* strain 2), and YMM (the carrier plus *S. cerevisiae* and half of the doses of *M. elsdenii* strain 1 and strain 2).

### Dual-Flow Continuous Culture System Operation

This experiment was performed using a dual-flow continuous culture system originally developed by Hoover et al. (1976) and successfully used for ruminal microbiome studies (Arce-Cordero et al., 2022a, 2022b; Monteiro et al., 2022b). The system was maintained at constant agitation (100 rpm), infusion of N_2_ gas, temperature (39 °C), and infusion of artificial saliva buffer with 0.40 g/L of urea (Weller and Pilgrim, 1974), at 3.05 mL per minute allowing for passage rates of 11% and 5.5% h^−1^ for liquid and solid effluents of digesta, respectively.

On the first day of each fermentation period, fermenters were inoculated with a pool of ruminal contents from three canulated Angus Steers (630 kg of BW) collected 2 h after feeding. From 2 wk before first collection and until the last collection, steers were maintained on a ration formulated with the same chemical composition as the non-acidotic diet provided to the fermenters on the first 8 d of fermentation. Ruminal contents were strained through four layers of cheesecloth, transferred into pre-warmed insulated jars, and immediately transported to the lab where each fermenter was pre-warmed and under continuous flush of N_2_ gas at the moment of inoculation with the mix of ruminal contents from the three steers. Throughout the experimental period, each fermenter was provided 107 g DM (dry matter) of the corresponding experimental diet per day, equally divided into two portions of 53.5 g DM at 0700 and 2100.

### Collection of Data and Samples

For each period, the first 5 d of fermentation were considered an adaptation for stabilization of bacterial communities (Salfer et al., 2018) and samples were collected on days 6, 7, and 8 (baseline non-acidotic conditions, before acidosis challenge, and supplementation of treatments) and on days 9,10, and 11 (during acidotic conditions and supplementation of treatments). Throughout sampling days, the containers of solid, and liquid digesta effluent were kept in an ice-cold water bath to prevent further digesta fermentation and preserve the quality of the samples.

Samples of liquid and solid digesta were collected every sampling days at 3, 6, and 9 h after morning feed provision and immediately stored at −80 °C for subsequent DNA (desoxyribonucleic acid) extraction for bacterial sequencing analysis. A total of 45 mL per fermenter per day were collected from the liquid effluent (15 mL at each timepoint). Conversely, 200 g of sample were collected from the solid effluent at each timepoint and strained through four layers of cheesecloth, totaling ~ 25 g of solid strained sample collected every day from each fermenter. Additionally, at the end of each sampling day, liquid, and solid effluents of each fermenter were combined and strained through four layers of cheesecloth for collection of a 10 mL sample which was acidified with 100 µL of 50% H_2_SO_4_ and stored at −20 °C for subsequent analyses of propionate.

### Laboratory Analyses

#### volatile fatty acids analysis.

Description of volatile fatty acids methodology can be found in our companion study (Monteiro et al., 2022a). Briefly, samples were centrifuged at 10,000 × *g* for 15 min and the supernatant was mixed with a solution of crotonic acid and metaphosphoric acid and frozen overnight. Samples were centrifuged again at 10,000 × *g* for 15 min and the supernatant was mixed with ethyl acetate, vortexed, and the top layer transferred to a chromatography injection vial for gas chromatography (Agilent 7820A GC, Agilent Technologies, Palo Alto, CA) with a flame ionization detector and a capillary column (CP-WAX 58 FFAP 25 m 0.53 mm, Varian CP7767, Varian Analytical Instruments, Walnut Creek, CA) maintained at 110 °C, with injector temperature at 200 °C, and detector at 220 °C. Propionate molar proportion was calculated by dividing propionate m*M* by total volatile fatty acids m*M*, and multiplying the result by 100, the final result was used for the correlation analysis of the current study.

#### DNA extraction.

All the samples that had been collected and stored at −80 °C were thawed at room temperature. Liquid and solid samples were processed independently. Samples of the same period, fermenter, and condition (non-acidotic or acidotic) were combined across days and timepoints, totaling 128 samples: 64 samples collected during non-acidotic conditions used as a baseline (32 from liquid effluent and 32 from solid effluent), and 64 samples collected during acidotic conditions used for evaluation of treatments effects (32 from liquid effluent and 32 from solid effluent). Extraction of genomic DNA was performed according to the methodology by Stevenson and Weimer (2007) previously described for samples collected from continuous culture fermenters (Arce-Cordero et al. 2022a, 2022b). Solid samples were processed by blending 22 g with extraction buffer (Tris HCl, ethylenediaminetetraacetic acid, and NaCl), the blend was centrifuged at 500 × *g* for 15 min at 4 °C, then 22 mL of supernatant were centrifuged at 10,000 × *g* for 25 min at 4 °C and the bacterial pellet was resuspended in DNA extraction buffer. For liquid samples, 22 mL were centrifuged at 10,000 × *g* for 25 min at 4 °C and the bacterial pellet was resuspended in DNA extraction buffer.

Bacterial pellets were mixed with 20% sodium lauryl sulfate solution and phenol, and disrupted with zirconium beads (BioSpec Products, Bartlesville, OK). Then, extraction of DNA was performed by sequential centrifugations with phenol, phenol/chloroform, and chloroform; then DNA was precipitated with 3 *M* Na acetate buffer and isopropanol. Finally, the DNA pellet was centrifuged with 70% ethanol and resuspended in Tris-EDTA buffer. The DNA concentration was measured using a Qubit Fluorometer (Invitrogen, San Diego, CA) and samples were stored at −80 °C.

#### DNA amplification and sequencing.

Amplification of the V4 hypervariable region of bacterial 16S rRNA (16S ribosomal ribonucleic acid) gene was performed with dual-index primers (Caporaso et al., 2011) according to Kozich et al. (2013). The following conditions were used for the PCR amplification reaction:1 µL forward index primer (10 m*M*), 1 µL reverse index primer (10 m*M*), 1 µL DNA template (10 ng/µL), 17 µL Pfx AccuPrime master mix (Invitrogen, USA), denaturation for 5 min at 95 ℃, 30 cycles of 95 ℃ for 30 s, annealing at 55 ℃ for 30 s, extension at 72 ℃ for 1 min, and elongation for 5 min at 72 ℃. Confirmation of PCR success was evaluated with PCR, and the resulting amplicons were run on a 1% agarose gel and normalized with a SequalPrep Normalization Plate Kit (Applied Biosystems, Foster City, CA) for preparation of the DNA pool library. Samples (*n* = 128) were sequenced using a MiSeq reagent kit V2 (2 × 250 cycles run; Illumina, San Diego, CA, USA) in an Illumina MiSeq platform (Illumina, San Diego, CA, USA) at the University of Florida’s Interdisciplinary Center for Biotechnology Research. Finally, the resulting sequencing data were deposited into the NCBI database with the following accession number PRJNA976746.

### Bacterial Sequence Data Analysis

Sequence data analysis was performed using Quantitative Insights into Microbial Ecology version 2 (QIIME 2) (Bolyen et al., 2019). Paired-end raw reads were imported and Interactive Quality Plot was used for evaluation of quality of the initial bases. Sequence quality control, filtering low-quality reads, denoising reads, merging paired-end reads, and removing chimeric reads were done with DADA2 pipeline implemented in QIIME 2. The phylogenetic tree was generated with align-to-tree-mafft-fasttree pipeline from the q2-phylogeny plugin of QIIME 2. Sequencing depth was normalized to 6,690 sequences per sample and the number of amplicon sequence variants, richness (Chao1), diversity (Shannon index), and Bray-Curtis distance were calculated by the core-metrics-phylogenetic method. Furthermore, amplicon sequence variants were classified by phylum, class, order, family, and genus, using the q2-feature-classifier plugin of QIIME 2 and the SILVA 138 database (https://www.arb-silva.de/documentation/release-1381/). Only average relative abundances greater than 0.1% were considered for analyses.

### Statistical Analysis

Bacterial community structure of bacteria during acidotic challenge was analyzed with Bray-Curtis distance of R vegan package (Callahan et al., 2016) and visualized with principal component analysis plots. Treatment differences in community structure and alpha diversity during acidotic challenge were analyzed with PERMANOVA using QIIME 2.

Relative abundance data of taxa were analyzed with the MIXED procedure of SAS 9.4 (SAS Institute Inc., Cary, NC). The statistical model included the fixed effect of treatment and random effects of period, square, and fermenter, and relative abundance during non-acidotic period was used as a covariate. Furthermore, the correlation between propionate molar proportion in ruminal fluid and relative abundance of taxa that were affected by treatments, was analyzed with Pearson correlation analysis. Significance threshold was declared at *P* ≤ 0.05, while 0.05 < *P* ≤ 0.10 was considered a trend.

## Result and Discussion

A total of 128 DNA samples were sequenced for the present study, which consisted of 64 samples collected during non-acidotic conditions (32 of liquid fraction and 32 of solid fraction) which were considered as the baseline, and 64 samples (32 of liquid fraction and 32 of solid fraction) that were collected during the period of acidotic conditions and treatment supplementation. As a result of 16S rRNA sequencing, a total of 6,449,196 reads were generated, which corresponded to 2,183,518 high-quality sequences that were retained for further analyses after filtering, denoising, merging, and removing chimeras with DADA2. Sequencing analysis allowed the identification of 11 phyla, 15 classes, 23 orders, 33 families, and 73 genera.

**Figure 1.**
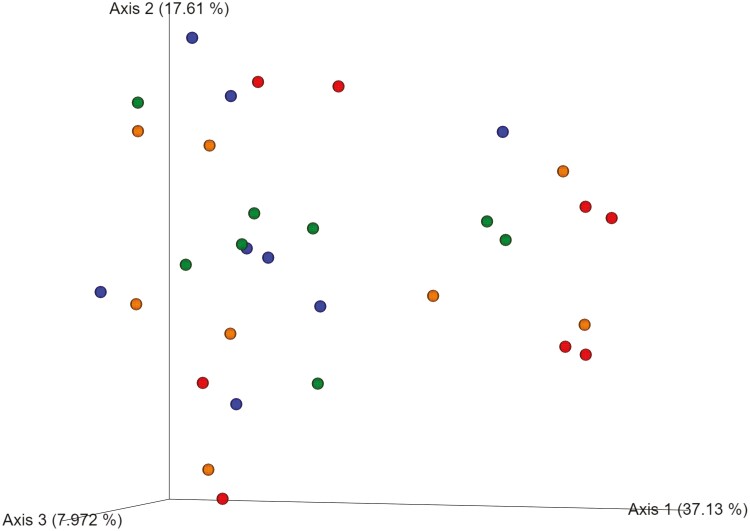
Principal coordinates analysis plots of Bray-Curtis similarity comparing the treatment effects on community structure of ruminal bacteria in liquid fraction during acidotic challenge. Treatments: CTRL, carrier of additives without direct-fed microbials (red); YM1, *Saccharomyces cerevisiae* and *Megasphaera elsdenii* strain 1 (blue); YM2, *Saccharomyces cerevisiae* and *Megasphaera elsdenii* strain 2 (orange); YMM, *Saccharomyces cerevisiae* and *Megasphaera elsdenii* strain 1 (1/2 dose) and strain 2 (1/2 dose) (green).

**Figure 2.**
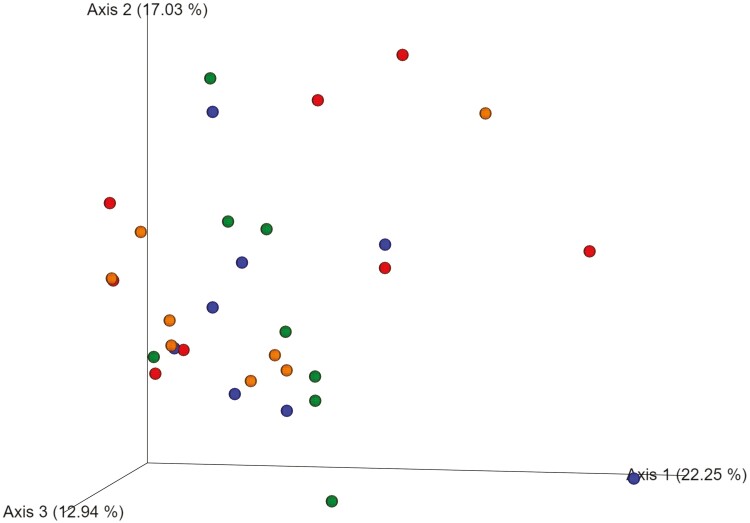
Principal coordinates analysis plots of Bray-Curtis similarity comparing the treatment effects on community structure of ruminal bacteria in solid fraction during acidotic challenge. Treatments: CTRL, carrier of additives without DFM (red); YM1, *Saccharomyces cerevisiae* and *Megasphaera elsdenii* strain 1 (blue); YM2, *Saccharomyces cerevisiae* and *Megasphaera elsdenii* strain 2 (orange); YMM, *Saccharomyces cerevisiae* and *Megasphaera elsdenii* strain 1 (1/2 dose) and strain 2 (1/2 dose) (green).

**Figure 3.**
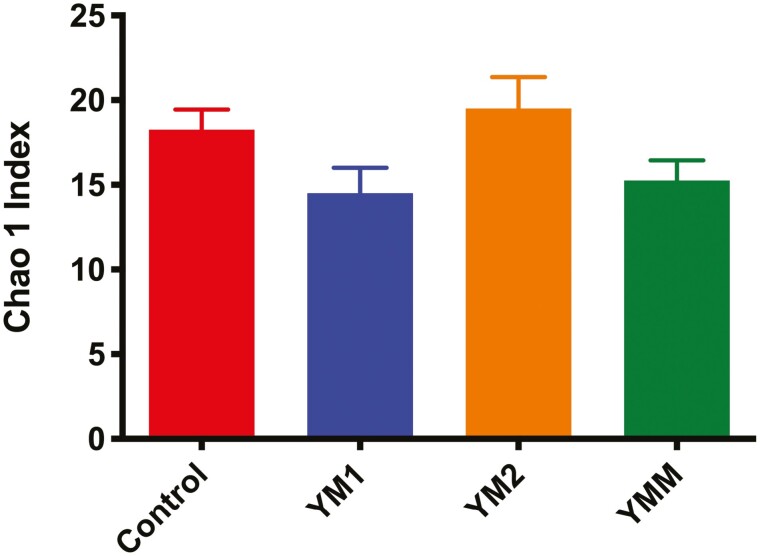
Effects of experimental treatments (*P* = 0.07) on alpha diversity (Chao 1 index) of ruminal bacteria in the solid fraction during acidotic challenge. Treatments: CTRL, carrier of additives without direct-fed microbials; YM1, *Saccharomyces cerevisiae* and *Megasphaera elsdenii* strain 1; YM2, *Saccharomyces cerevisiae* and *Megasphaera elsdenii* strain 2; YMM, *Saccharomyces cerevisiae* and *Megasphaera elsdenii* strain 1 (1/2 dose) and strain 2 (1/2 dose).

**Figure 4.**
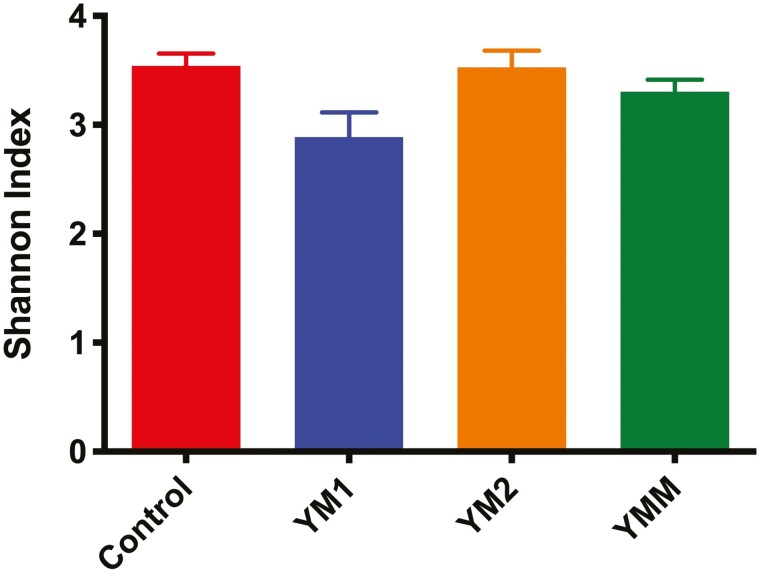
Effects of experimental treatments (*P* = 0.02) on alpha diversity (Shannon index) of ruminal bacteria in the solid fraction during acidotic challenge. Treatments: CTRL, carrier of additives without direct-fed microbials; YM1, *Saccharomyces cerevisiae* and *Megasphaera elsdenii* strain 1; YM2, *Saccharomyces cerevisiae* and *Megasphaera elsdenii* strain 2; YMM, *Saccharomyces cerevisiae* and *Megasphaera elsdenii* strain 1 (1/2 dose) and strain 2 (1/2 dose).

Figures 1 and 2 summarize the effects of DFM on bacterial community structure during an acidotic challenge, in the liquid and solid fractions, respectively. As noted in the figures, based on Bray-Curtis similarity index, DFM did not affect bacterial community structure of either of the fractions analyzed. For bacterial alpha diversity, we found an effect of DFM on richness and diversity in the solid fraction. Richness tended to be lower for YM1 in comparison to control and YM2 as indicated by the Chao 1 index (Figure 3). Moreover, Shannon index (Figure 4) was lower in YM1 than in control and YM2, indicating a reduction of bacterial diversity for YM1 during the acidotic challenge compared to the other treatments. Such a decrease in bacterial diversity in YM1 suggests that YM1 treatment favored specific taxonomic groups over others during the acidotic conditions of the experiment.

We evaluated the effects of DFM on microbial relative abundance at the phylum, family, and genera levels in both liquid and solid fractions. Similar to our results for alpha diversity, at the phylum level (Table 2) we only found DFM effects in the solid fraction, where YM1 had a greater relative abundance of *Proteobacteria* than control, which seemed to be accompanied by an opposite change in relative abundance of *Firmicutes* that tended to be lower in YM1 than control.

**Table 2.** Effects of direct-fed microbials on phylum relative abundance of bacteria in liquid and solid fractions during acidotic challenge

Phylum	Treatment means^1^				SEM	*P*-value^2^
	Control	YM1	YM2	YMM		
Liquid fraction						
* Bacteroidota*	43.6	33.5	39.6	40.1	4.81	0.46
* Proteobacteria*	26.9	42.3	32.8	35.8	6.57	0.12
* Firmicutes*	27.1	23.7	25.3	22.0	3.28	0.41
Solid fraction						
* Bacteroidota*	39.4	37.6	36.5	36.5	3.50	0.92
* Firmicutes*	35.0	24.6	34.1	31.0	3.24	0.10
* Proteobacteria*	25.6^b^	37.1^a^	28.8^ab^	31.4^ab^	3.29	0.05

^1^Treatments based on direct-fed microbials (DFM) supplementation: CTRL, carrier of additives without DFM; YM1, *Saccharomyces cerevisiae* and *Megasphaera elsdenii* strain 1; YM2, *Saccharomyces cerevisiae* and *Megasphaera elsdenii* strain 2; YMM, *Saccharomyces cerevisiae* and *Megasphaera elsdenii* strain 1 (1/2 dose) and strain 2 (1/2 dose).

^2^Effect of treatment. Treatment means with a different superscript within the same row are statistically different from each other.

Our results show that under acidotic challenge conditions promoted by a high-grain diet, between 99% and 100% of the sequences at the phylum level are represented by only three phyla. More specifically, average relative abundance of phyla across treatments in the liquid fraction was 39.2%, 34.5%, and 24.5% for *Bacteroidota*, *Proteobacteria*, and *Firmicutes*, respectively. On the other hand, for the solid fraction, *Bacteroidota* also was the most abundant phylum, followed by *Firmicutes* and *Proteobacteria*, with average relative abundances of 37.5, 31.2%, and 30.7%, respectively. Importance of *Bacteroidota* phylum in starch digestion and therefore high-grain diets, has been previously demonstrated by Deusch et al. (2017) who evaluated diets with increasing inclusion of starch and identified 52 proteins involved in propionate synthesis, of which 25 enzymes were synthesized by bacteria within phylum *Bacteroidota*, which were more abundant in the diet with highest starch concentration.

Previous studies evaluating microbial populations in continuous cultures of ruminal contents have reported a greater number of phyla representing a similar share of the total sequences. In a study evaluating diets with 30% to 40% neutral detergent fiber (NDF) Arce-Cordero et al. (2022a) found the six most abundant phyla represented ~96% of total sequences. Similarly, Monteiro et al. (2022b), found that almost 97% of total sequences were represented by the most abundant seven phyla when diets with 32% NDF were evaluated in continuous culture fermenters. However, such studies evaluated diets for dairy cows with a greater NDF concentration and lower supply of starch, therefore imposing a lower acidogenic potential compared to our current study.

Average pH in our current study ranged between 5.2 and 5.8 as reported in our companion paper (Monteiro et al., 2022a), with the lower values corresponding to the days when the acidotic challenge was imposed. Conversely, average daily pH in Arce-Cordero et al. (2022a) was 6.3 and ranged between 5.8 and 6.8 in Monteiro et al. (2022b). Our findings in the present study are consistent with findings by Plaizier et al. (2017a, 2017b) showing a lower microbial diversity in the rumen of animals fed diets that promote a lower ruminal pH. Interestingly, DFM inclusion in the diet, more specifically the YM1 treatment, further reduced the already low microbial diversity imposed by the acidotic challenge, as indicated by results of Chao 1 (Figure 3) and Shannon index (Figure 4), which together with results of phyla relative abundance (Table 2) indicate that YM1 affected microbial diversity in the solid fraction at least partly by favoring relative abundance of *Proteobacteria* at the expense of *Firmicutes*.

Further analysis at lower taxonomic levels revealed that YM1 tended to increase relative abundance of *Succinivibrionaceae* family in the liquid fraction (Table 3), while in the solid fraction the relative abundance of the same family was greater for YM1 compared to control. Conversely, abundance of family *Lachnospiraceae* was lower for YM1 than Control and YM2 in solid fraction. Such an increase in *Succinivibrionaceae* explains the greater relative abundance of phylum *Proteobacteria* resulting from YM1 inclusion in the diet. Similarly, a lower abundance of *Lachnospiraceae*, which belongs to phylum *Firmicutes*, is consistent with a lower abundance of bacteria in this phylum with YM1.

**Table 3.** Effects of direct-fed microbials on family relative abundance of bacteria in liquid and solid fractions during acidotic challenge

Family	Treatment means^1^				SEM	*P*-value^2^
	Control	YM1	YM2	YMM		
Liquid fraction						
* Prevotellaceae*	43.3	31.8	38.5	40.2	0.82	0.34
* Succinivibrionaceae*	26.9	42.5	32.8	35.6	6.52	0.10
* Selenomonadaceae*	12.2	11.3	12.1	9.56	2.62	0.72
* Acidaminococcaceae*	7.55	5.05	4.44	4.59	2.73	0.39
Solid fraction						
* Prevotellaceae*	38.9	37.2	35.2	36.3	3.50	0.91
* Succinivibrionaceae*	26.0^b^	37.1^a^	28.4^ab^	31.6^ab^	3.30	0.05
* Selenomonadaceae*	15.5	10.2	12.6	12.0	2.83	0.59
* Lachnospiraceae*	13.4^a^	8.21^b^	15.0^a^	11.1^ab^	1.66	0.04

^1^Treatments based on direct-fed microbials (DFM) supplementation: CTRL, carrier of additives without DFM; YM1, *Saccharomyces cerevisiae* and *Megasphaera elsdenii* strain 1; YM2, *Saccharomyces cerevisiae* and *Megasphaera elsdenii* strain 2; YMM, *Saccharomyces cerevisiae* and *Megasphaera elsdenii* strain 1 (1/2 dose) and strain 2 (1/2 dose).

^2^Effect of treatment. Treatment means with a different superscript within the same row are statistically different from each other.

At the genus level (Table 4), we found a trend for a greater relative abundance of *Succinivibrio* in the liquid fraction of YM1 and YMM compared to Control and YM2. Such results explain the trend towards a greater relative abundance of *Succinivibrionaceae* family observed in the liquid fraction. Conversely, in the solid fraction, although we observed a numerical increase in *Succinivibrionaceae_UCG-001* and *Succinivibrio* for YM1 compared to Control, such increases were not statistically significant. However, the relative abundance of *Oribacterium* in the solid fraction tended to be lower for YM1 in comparison to the three other treatments. Since the genus *Oribacterium* belongs to phylum *Firmicutes* and family *Lachnospiraceae*, a lower abundance of *Oribacterium* is consistent with our results at the phylum and family level in the solid fraction.

**Table 4.** Effects of direct fed microbials on genus relative abundance of bacteria in liquid and solid fractions during acidotic challenge

Genus	Treatment means^1^				SEM	*P*-value^2^
	Control	YM1	YM2	YMM		
Liquid fraction						
* Prevotella*	42.0	29.5	37.0	39.6	5.00	0.24
* Succinivibrionaceae_UCG-001*	23.8	37.7	28.8	26.8	5.16	0.13
* Uncultured selenomonadaceae*	4.74	6.62	6.64	4.67	2.84	0.75
* Succinivibrio*	3.30	5.06	3.52	8.96	2.40	0.09
* Succiniclasticum*	6.72	5.06	4.46	4.11	2.70	0.63
* Unclassified selenomonadaceae*	6.63	1.90	4.49	3.21	2.09	0.22
Solid fraction						
* Prevotella*	35.5	36.5	34.4	35.6	3.21	0.98
* Succinivibrionaceae_UCG-001*	21.5	29.6	24.5	23.1	5.50	0.48
* Oribacterium*	7.24	4.35	6.73	7.38	0.92	0.10
* Succinivibrio*	4.07	8.13	3.37	8.24	3.45	0.32
* Succiniclasticum*	4.29	3.06	7.12	7.13	1.75	0.19
* Unclassified selenomonadaceae*	7.47	1.30	6.23	4.69	2.67	0.34

^1^Treatments based on direct fed microbials (DFM) supplementation: CTRL, carrier of additives without DFM; YM1, *Saccharomyces cerevisiae* and *Megasphaera elsdenii* strain 1; YM2, *Saccharomyces cerevisiae* and *Megasphaera elsdenii* strain 2; YMM, *Saccharomyces cerevisiae* and *Megasphaera elsdenii* strain 1 (1/2 dose) and strain 2 (1/2 dose).

^2^Effect of treatment.

**Figure 5.**
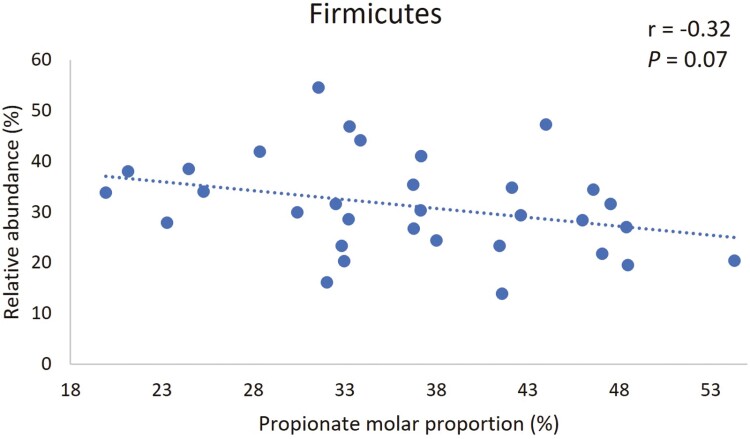
Correlation between molar proportion of propionate in fluid and relative abundance of phylum *Firmicutes* in solid fraction during acidotic challenge.

**Figure 6.**
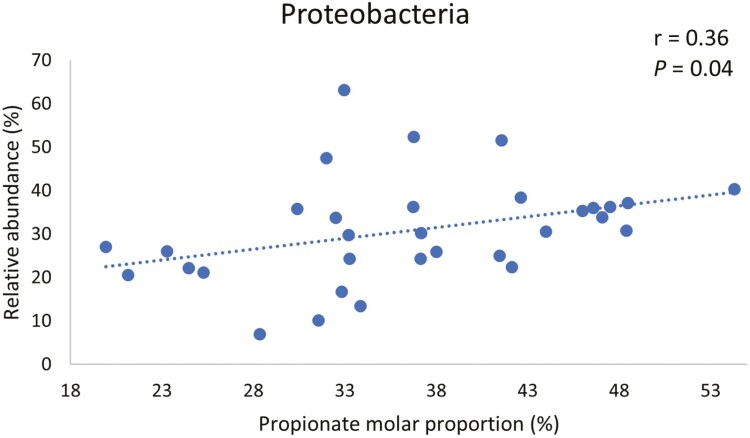
Correlation between molar proportion of propionate in fluid and relative abundance of phylum *Proteobacteria* in solid fraction during acidotic challenge.

**Figure 7.**
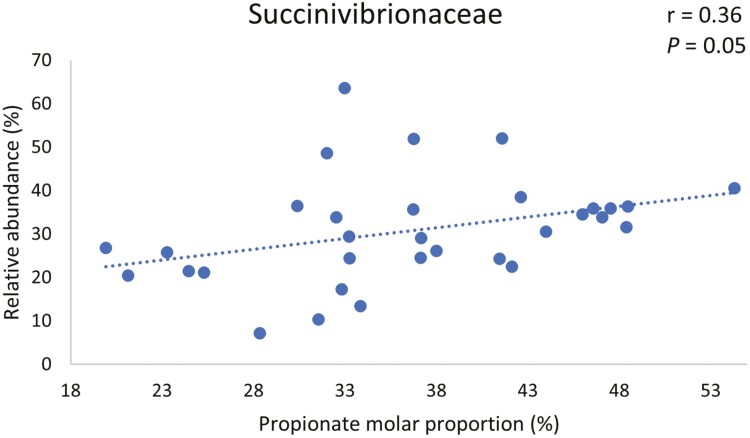
Correlation between molar proportion of propionate in fluid and relative abundance of family *Succinivibrionaceae* in solid fraction during acidotic challenge.

Based on our results of the effects of DFM on taxa relative abundance in the current study, and the results of our companion study (Monteiro et al., 2022a) where we found an increase in propionate molar proportion resulting from DFM inclusion in diets, we performed a correlation analysis to evaluate the association between propionate molar proportion and relative abundance of taxa that were affected by DFM during the acidotic challenge. We found that molar proportion of propionate tended to be negatively correlated to relative abundance of bacteria of the phylum *Firmicutes* in the solid fraction (Figure 5). Conversely, relative abundances of phylum *Proteobacteria* and family *Succinivibrionaceae* (which belongs to phylum *Proteobacteria*), were positively correlated to molar proportion of propionate as shown in Figures 6 and 7, respectively.

Altogether, our results indicate that inclusion of DFM in the diets with high acidogenic potential, and more specifically inclusion of YM1, favors propionate synthesis as shown by a greater propionate molar proportion, which is consistently associated with greater relative abundance of bacterial taxa involved in ruminal synthesis of succinate, an intermediary in the randomizing pathway of propionate synthesis (Gylswyk, 1995; Ungerfeld, 2015). A greater molar proportion of propionate resulting from YM1 would be expected since one of the microbial components of YM1 is *M. elsdenii*, a lactate-utilizing bacterium that synthesizes propionate. However, in the present study, YM1 is favoring relative abundance of bacteria involved in a different pathway of propionate synthesis (succinate pathway) which does not utilize lactate as a substrate.

The fact that our treatments are combinations of DFM implies that a greater molar proportion of propionate and a greater relative abundance of succinate synthesizing bacteria could be facilitated by either of the components of YM1 (*S. cerevisiae* and *M. elsdenii* strain 1). Lactate utilization through the acrylate pathway is an option that we cannot confirm since we did not find any effect of our treatments on lactate concentrations in our companion study (Monteiro et al., 2022a). The yeast component of the treatment may also have played a role in the effects of YM1 on propionate synthesis. Many studies have associated yeast supplementation in ruminants with a stimulatory effect of fiber-degrading bacteria without reporting a direct effect on propionate synthesis (Desnoyers et al., 2009; Oh et al., 2019). However, other studies have reported a greater concentration of propionate resulting from yeast supplementation in ruminants (Guedes et al., 2008; Pinloche et al., 2013; AlZahal et al., 2014).

Ogunade et al. (2019) found that yeast supplementation increased relative abundance of ruminal bacteria involved in propionate synthesis in beef cattle. The resulting increase in propionate and consequently a lower acetate to propionate ratio may play a role in animal response to yeast supplementation. According to Baker et al. (2022), the differential response in ruminal propionate synthesis across yeast supplementation studies may be a consequence of the type of diet fed or the dose fed. The type of diet in our study with a high concentration of starch, may have favored the response on propionate synthesis and succinate synthesizing bacteria found in our study. Gao and Geng (2022) found that active dry yeast supplementation to finishing bulls led to a shift in the most abundant bacterial genus in the rumen from *Ruminobacter* to *Succiniclasticum*, which the authors mention as a possible factor contributing to ruminal pH stabilization in beef cattle consuming high-grain diets.

## Conclusions

In conclusion, our results indicate that the addition of YM1 (*S. cerevisiae* and *M. elsdenii* strain 1) under acidotic conditions promotes a greater synthesis of propionate in continuous culture of ruminal contents, which may be driven by a greater relative abundance of bacteria involved in synthesis of succinate that increase availability of succinate and may favor propionate synthesis through the randomizing pathway.
